# Matched Analyses of Brain Metastases versus Primary Non-Small Cell Lung Cancer Reveal a Unique microRNA Signature

**DOI:** 10.3390/ijms24010193

**Published:** 2022-12-22

**Authors:** Georgios Tsakonas, Andreas Koulouris, Dominika Kazmierczak, Johan Botling, Cristian Ortiz-Villalon, Helena Nord, Magnus Lindskog, Martin Sandelin, Patrick Micke, Per Hydbring, Simon Ekman

**Affiliations:** 1 Thoracic Oncology Center, Karolinska University Hospital, 17176 Stockholm, Sweden; 2 Department of Oncology and Pathology, Karolinska Institutet, 17164 Stockholm, Sweden; 3 Department of Immunology, Genetics and Pathology, Uppsala University, 75105 Uppsala, Sweden; 4 Department of Immunology, Genetics and Pathology and Science for Life Laboratory, Uppsala University, 75105 Uppsala, Sweden; 5 Department of Pelvic Cancer, Genitourinary Oncology Unit, Karolinska University Hospital, 17176 Stockholm, Sweden; 6 Department of Medical Sciences, Department of Oncology, University Hospital, Uppsala University, 75185 Uppsala, Sweden

**Keywords:** non-small cell lung cancer, brain metastasis, microRNA

## Abstract

Distant spreading of tumor cells to the central nervous system in non-small cell lung cancer (NSCLC) occurs frequently and poses major clinical issues due to limited treatment options. RNAs displaying differential expression in brain metastasis versus primary NSCLC may explain distant tumor growth and may potentially be used as therapeutic targets. In this study, we conducted systematic microRNA expression profiling from tissue biopsies of primary NSCLC and brain metastases from 25 patients. RNA analysis was performed using the nCounter Human v3 miRNA Expression Assay, NanoString technologies, followed by differential expression analysis and in silico target gene pathway analysis. We uncovered a panel of 11 microRNAs with differential expression and excellent diagnostic performance in brain metastasis versus primary NSCLC. Five microRNAs were upregulated in brain metastasis (miR-129-2-3p, miR-124-3p, miR-219a-2-3p, miR-219a-5p, and miR-9-5p) and six microRNAs were downregulated in brain metastasis (miR-142-3p, miR-150-5p, miR-199b-5p, miR-199a-3p, miR-199b-5p, and miR-199a-5p). The differentially expressed microRNAs were predicted to converge on distinct target gene networks originating from five to twelve core target genes. In conclusion, we uncovered a unique microRNA profile linked to two target gene networks. Our results highlight the potential of specific microRNAs as biomarkers for brain metastasis in NSCLC and indicate plausible mechanistic connections.

## 1. Introduction

Lung cancer is still the leading cause of cancer-related mortality globally [1]. Non-small cell lung cancer (NSCLC) with brain metastases (BM) is a frequent and challenging clinical problem, especially in patients with adenocarcinoma and oncogenic driven tumors [2,3,4]. Patients with BM have worse prognosis. With the exception of patients with oncogenic driven tumors that respond well to targeted therapy [5,6], the response rates to systemic therapy are generally lower in non-oncogenic-driven NSCLC patients with brain metastatic disease, albeit substantial responses have been observed in some patients receiving platinum-doublet chemotherapy or combination immunotherapy regimens [7,8,9,10]. Systemic treatment with single PD-1/PDL-1 inhibitors has shown limited efficacy in the treatment of brain metastatic NSCLC, a fact that is likely related to the unique tissue properties of the central nervous system (CNS), and the clonal selection and clonal evolution of the cancer cells that disseminate to the brain during the course of the disease [11,12].

MicroRNAs (miRNAs) are pleiotropic, non-coding, small RNAs that play an important role in cancer growth, invasion, metastasis, angiogenesis, and apoptosis through the regulation of the expression of coding RNAs [13,14]. miRNAs can function both as tumor suppressors and tumor promoters, depending on the gene pathways affected. A single miRNA can target up to several hundred mRNAs by binding to their 3′-untranslated regions resulting in mRNA degradation or the inhibition of protein translation [15,16]. Some miRNAs have also been proposed as promoters of drug resistance in NSCLC patients receiving systemic therapy [17]. Moreover, miRNAs have been suggested as biomarkers to predict BM in cancer, including in NSCLC [18,19].

We conducted gene expression analysis from formalin-fixed, paraffin-embedded (FFPE) tissue sections of primary lung tumors and matched BM. The aim of our study was to explore the differential expression of miRNAs between the primary tumor and BM, and analyze the molecular pathways that could be affected by a potential up- or downregulation of miRNAs.

## 2. Results

### 2.1. Patient Characteristics

Patient characteristics including demographic variables and clinical aspects of the whole cohort are included in Table 1. The most common sex and histology were male (64%) and adenocarcinoma (68%), respectively. None of the patients received systemic treatment as a first-line option for CNS disease. The most frequent recursive-partitioning analysis (RPA) and graded prognostic assessment (GPA) groups were 2 for both; 64% and 56%, respectively [12]. The univariate Cox regression analysis for demographics and clinical variables showed that sex, BM at diagnosis, RPA class, PS, and number of BM had a prognostic value [12].

### 2.2. Gene Expression Analysis

**Figure 1:**
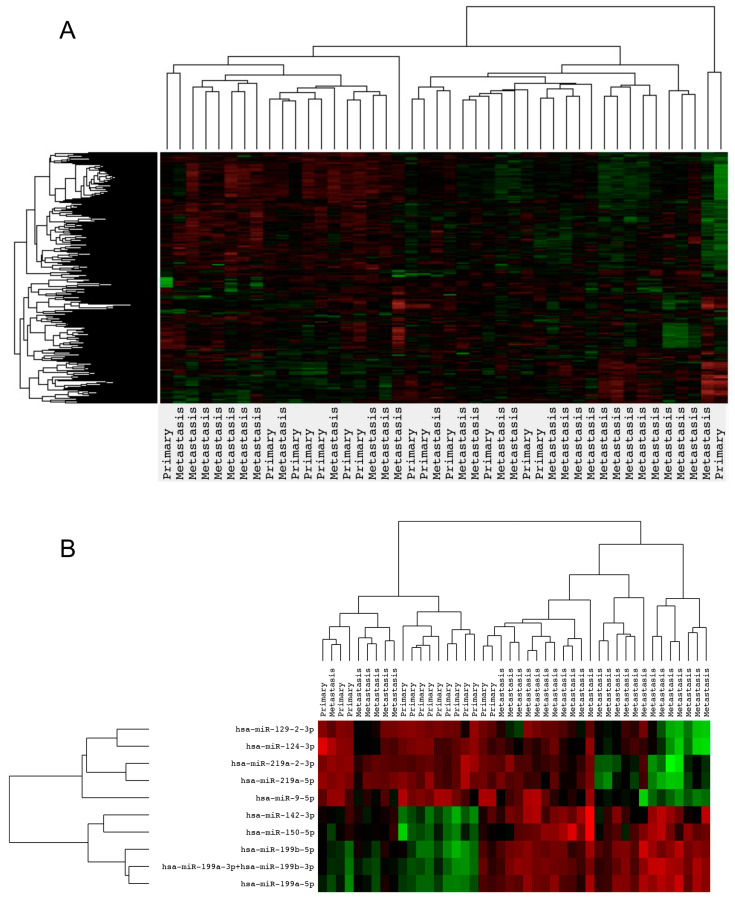
Hierarchical clustering of miRNAs in primary NSCLC versus brain metastasis. (**A**) Unsupervised hierarchical clustering of 827 human miRNAs in primary NSCLC versus brain metastatic lesions. (**B**) Clustering of differentially expressed miRNAs in primary NSCLC versus brain metastatic lesions. Rows display individual miRNAs; columns display sample source. Color scale is depicted from red to green. Red = low expression; green = high expression.

Our lung cancer cohort contained 44 samples separated into 14 primary specimens and 30 brain metastatic specimens originating from 25 patients. Total RNA was extracted from all samples, adjusted to equal amounts, and loaded onto the NanoString Human v3 miRNA Assay panel containing probes for 827 human miRNAs. Differential expression was conducted by analyzing primary samples versus brain metastatic samples and ranked according to statistical significance with *p*-values corrected for multiple testing (FDR < 0.05). This revealed statistically significant differential signals between the two groups for ten miRNA probes covering eleven miRNAs. Five miRNAs were upregulated in brain metastatic samples (miR-9-5p, miR-129-2-3p, miR-124-3p, miR-219a-5p, and miR-219a-2-3p) and six miRNAs were downregulated (miR-199a-5p, miR-199a-3p, miR-199b-3p, miR-199b-5p, miR-150-5p, and miR-142-3p) in brain metastatic samples (Table S1). The relatively low number of differentially expressed miRNAs was reflected when plotting the entire array for unsupervised hierarchical clustering, resulting in an equal distribution of primary and metastatic samples between the major clusters (Figure 1A). When performing hierarchical clustering with only differentially expressed miRNAs (FDR < 0.05) as input, brain metastatic samples were clearly separated from primary samples. The two major sample clusters contained a total of 18 and 26 samples, respectively, with a distribution of brain metastatic samples in the two clusters of 6 out of 18 samples and 24 out of 26 samples, respectively (Figure 1B). Of note, four out of six downregulated miRNAs in brain metastatic samples belonged to the miR-199 family, while two out of five upregulated miRNAs in brain metastatic samples belonged to the miR-219 family (Figure 1B).

**Figure 2:**
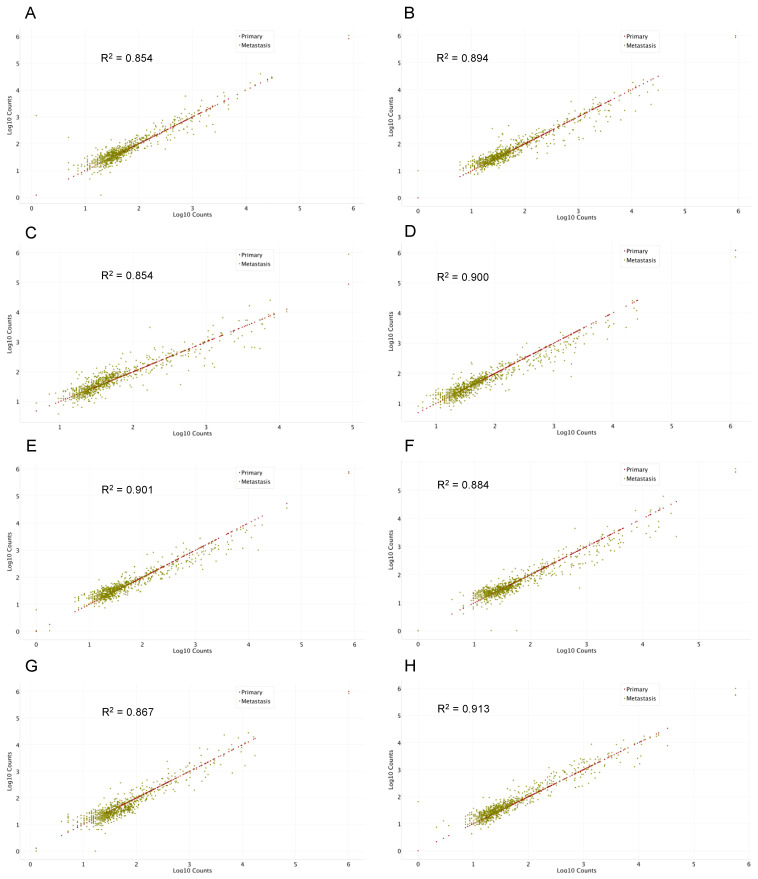
Correlation scatter plot of miRNA expression (log 10 counts) in primary NSCLC versus brain metastasis. (**A**–**H**) Individual examples of global miRNA expression (log 10 counts) in paired primary versus brain metastatic NSCLC. Each dot represents one miRNA. miRNAs in primary NSCLC are plotted on a linear line with identical expression on the *x*- and *y*-axis. miRNAs in brain metastasis are plotted either above or below the corresponding miRNA in primary NSCLC with expression indicated on the *y*-axis. Red = miRNA expression in primary NSCLC; green = miRNA expression in metastatic lesions. R-squared values of least squares regression are indicated in all panels for miRNAs expressed in metastatic lesions.

In line with the outcome of the analysis of differential gene expression, individual patient analysis of paired primary and brain metastatic samples did not reveal any global alteration in miRNA expression through scatter plot analysis (Figure 2A–H). Moreover, the differentially expressed miRNAs from the full cohort were following the identical trend in individually paired samples (Figure S1A–J, Table S2).

**Figure 3:**
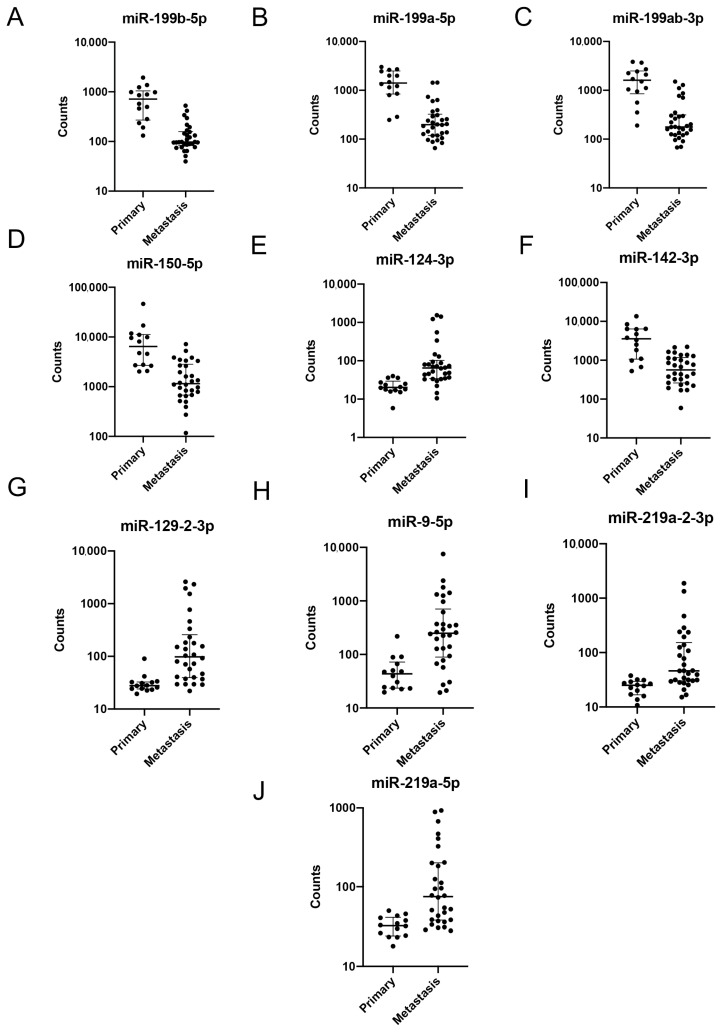
Expression of differentially expressed miRNAs in primary NSCLC versus brain metastasis. (**A**–**J**) Normalized counts of differentially expressed miRNAs comparing primary NSCLC versus brain metastasis. All displayed miRNAs were differentially expressed with a false discovery rate adjusted *p*-value of <0.05. miRNAs from the miR-199 family displayed the most significant differential expression.

When analyzing absolute expression of the differentially expressed miRNAs, we noted a substantial range in endogenous expression. All members of the miR-199 family as well as miR-150-5p and miR-142-3p were abundantly expressed in both the primary and in the brain metastatic samples while miR-124-3p, miR-129-2-3p, miR-9-5p, miR-219a-5p, and miR-219a-2-3p displayed counts close to background level in the primary sample (Figure 3A–J).

**Figure 4:**
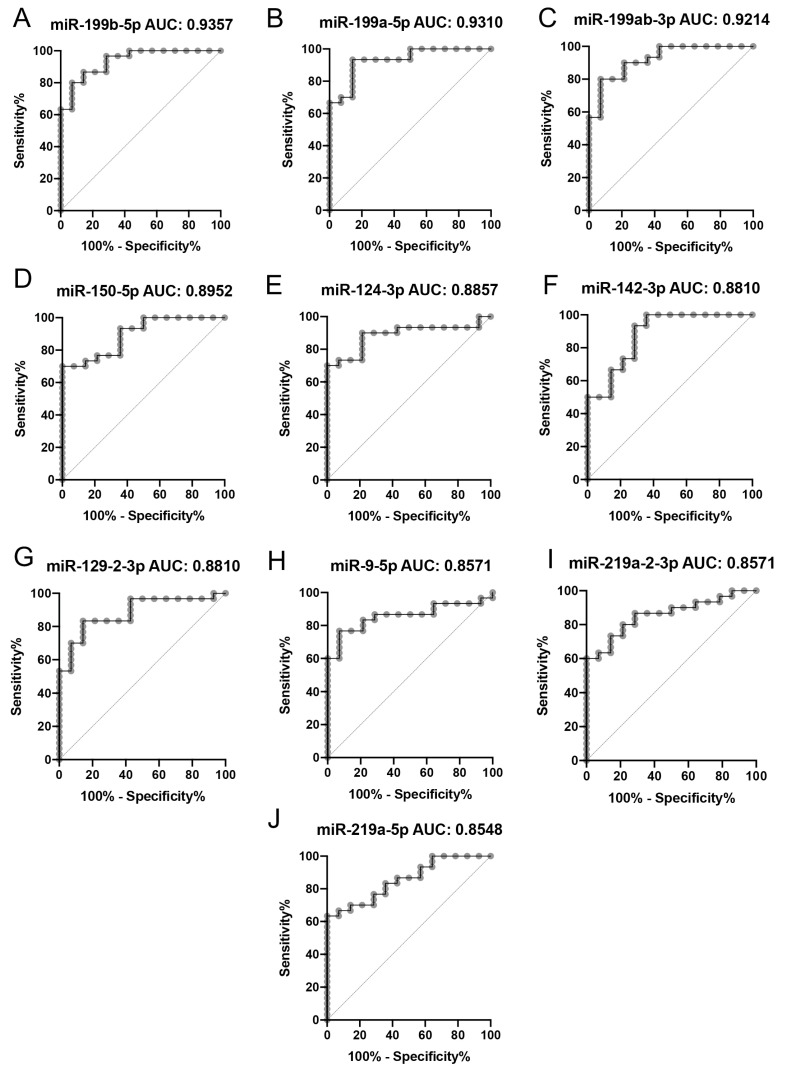
Receiving operating characteristics analysis of differentially expressed miRNAs in primary NSCLC versus brain metastasis. (**A**–**J**) Individual miRNAs are displayed with declining area under the curve (AUC) values. miRNAs from the miR-199 family displayed the highest diagnostic potential.

The absolute expression of the differentially expressed miRNAs correlated with their diagnostic potential, displayed by area under curve (AUC) values in receiver operating characteristics (ROC) analysis (Figure 4A–J). The miR-199 miRNA family members, which were all abundantly expressed in both samples, displayed the highest AUC values (0.9214–0.9357) of all miRNAs while the lowest AUC score was computed for miR-219a-5p (0.8548) (Figure 4A–J).

### 2.3. Pathway Analysis

**Figure 5:**
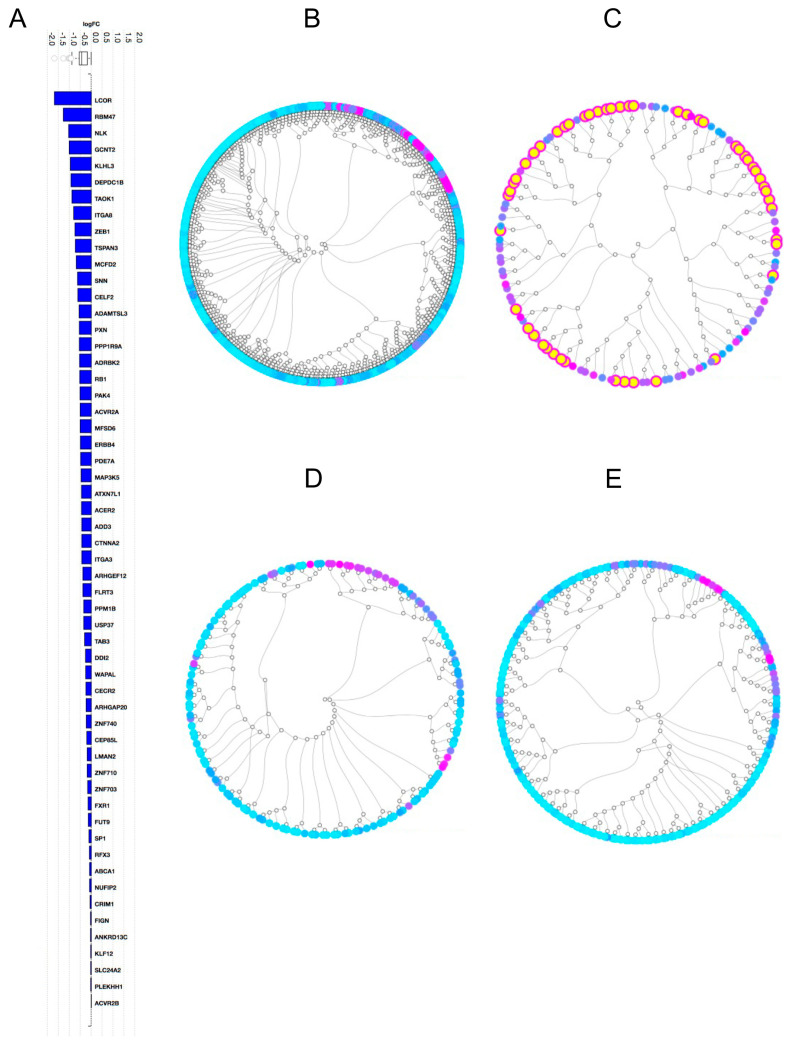
In silico predicted target gene analysis and pathway analysis using the iPathwayGuide. (**A**) Top downregulated target genes using all differentially expressed miRNAs; (**B**–**E**) dendrograms of all target genes using all differentially expressed miRNAs displaying biological processes (**B**); pathways (**C**); molecular functions (**D**); and cellular components (**E**). Each circle in the outer ring displays one entity, i.e., one biological process, pathway, molecular function, or cellular component. Inner lines and circles display connections between the different entities. Highlighted entities in panel C display the top 50 most statistically significant pathways. Overall statistical significance is displayed as a color range from green (less significant) to purple (more significant).

In an attempt to decipher the molecular impact of our differentially expressed miRNAs, we turned to in silico analysis using the iPathway software from Advaita Bioinformatics. First, we extracted all predicted target genes (6028 genes) of our 11 differentially expressed miRNAs from TargetScan 7 and ranked them according to their cumulative context score (>0.01) (Table S3). Next, we transformed the context scores into statistically significant logFC values of each gene by combining the number of events for each gene with the number of links to a miRNA that was differentially expressed. Target genes linked to miRNAs solely upregulated or solely downregulated in brain metastasis would receive a bigger logFC value compared with target genes linked to multiple miRNAs either being up- or downregulated in brain metastasis. When analyzing the outcome of all target genes from all differentially expressed miRNAs, a variety of gene mRNA transcripts were displayed with the mRNA of *LCOR* (Ligand-dependent nuclear receptor corepressor) as the most pronounced candidate (Figure 5A). Furthermore, by using all target genes from all differentially expressed miRNAs, we analyzed dendrograms of biological processes (Figure 5B), pathways (Figure 5C), molecular functions (Figure 5D), and cellular components (Figure 5E). We did not identify specific molecular pathways of more substantial biological significance than others (Table S4).

**Figure 6:**
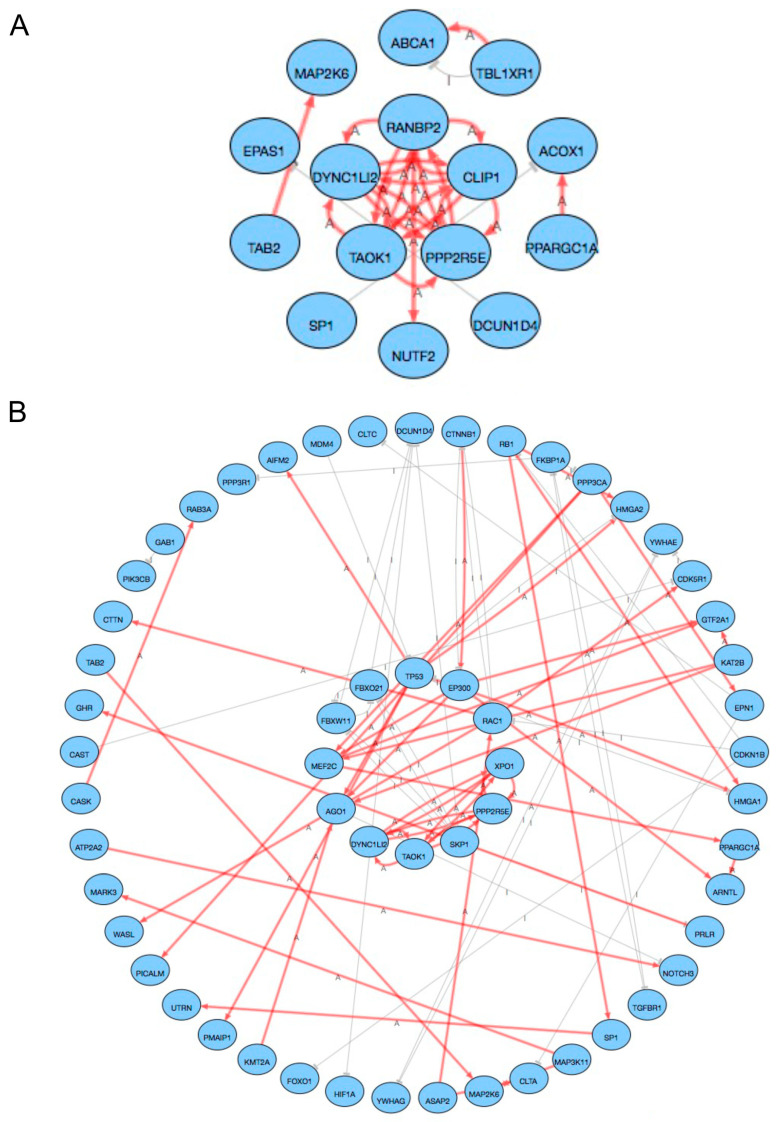
Hub gene networks of in silico predicted miRNA target genes. (**A**) Network of target genes related to miRNAs upregulated in BM. (**B**) Network of target genes related to miRNAs downregulated in BM. Connections marked “A” display activation. Connections marked “I” display inhibition.

Therefore, we turned to the network analysis function in the iPathway software. This function allowed us to look at the target genes of specific miRNAs and filter out all target genes that failed to display a minimum of one regulatory connection, i.e., activation or inhibition of another target gene (Figure 6). We performed two separate analyses, target genes with regulatory connections to other target genes of miRNAs being upregulated in brain metastasis (Figure 6A), and target genes with regulatory connections to other target genes of miRNAs being downregulated in brain metastasis (Figure 6B). For miRNAs being upregulated in brain metastasis, our network analysis revealed a core network of five target genes (*RANBP2*, *CLIP1*, *PPP2R5E*, *TAOK1*, and *DYNC1LI2*) predicted to be downregulated in brain metastasis (Figure 6A). For miRNAs being downregulated in brain metastasis, our network analysis revealed a core network of twelve target genes (*TP53*, *EP300*, *RAC1*, *XPO1*, *PPP2R5E*, *SKP1*, *TAOK1*, *DYNC1LI2*, *AGO1*, *MEF2C*, *FBXW11*, and *FBXO21*) predicted to be upregulated in brain metastasis (Figure 6B). For the predicted downregulated targets in brain metastasis, we found most regulatory connections, eight connections, for *CLIP1* (Figure 6A). For the predicted upregulated targets in brain metastasis, we found most regulatory connections, nine connections, for *TP53* (Figure 6B). Finally, we turned to TarBase v.8, an online tool, for experimentally verified miRNA targets. Applying all the differentially expressed miRNAs with all of the core hub target genes generated 22 interactions in the published literature, linking three differentially expressed miRNAs to core hub target genes for all targets except *SKP1* (Table S5).

## 3. Discussion

The results of our analyses of the differential expression of miRNAs between the primary tumor and the CNS compartment, as well as its effect on cellular biological functions, can be divided into two major parts. In the first part of our study, we found that some miRNAs were upregulated or downregulated in the BM compared with the primary tumor, which is something that could eventually lead to molecular alterations on the transcriptional level of coding RNAs. Two of the five upregulated miRNAs in the BM belong to the miR-219 family. miR-219 can decrease cell proliferation and epithelial mesenchymal transition (EMT) through the silencing of *XB130* in cell cultures, albeit its role in vivo is not yet fully understood [20]. Another reported in vitro target of miR-219 is the high mobility group AT-hook 2 (*HMGA2*). The downregulation of *HMGA2* also leads to decreased cell proliferation and EMT [21]. On the other hand, miR-9-5p, which was also found to be upregulated in BM, promotes cancer cell progression by silencing *ID4* in lung adenocarcinoma cell lines [22]. A higher concentration of miR-9-5p in NSCLC tissue biopsies has also been shown to suppress *TGFBR2*, which induces cell proliferation, invasion, and metastasis [23]. A study on pemetrexed-resistant lung cancer cell lines showed that high concentration of miR-124-3p, which was upregulated in BMs in our study, can decrease pemetrexed resistance by inhibiting the FGF2–EGFR pathway [24]. miR-124-3p achieves this inhibition by targeting N-acetylglucosaminyltransferase V (*MGAT5*), a correlation which has been shown in in vitro and in vivo breast cancer mouse models [25]. Bioengineered miR-124-3p suppressed the expression of multiple proteins, which are critical for cancer invasion and metastasis, in a proteomics study done in lung cancer cell lines and in an experimental metastasis mouse model. The proteins, which were downregulated, affected the cytoskeleton, cellular junction, and cellular adhesion [26]. Zinc finger E-box binding homeobox 2 (*ZEB2*), a regulator of EMT, as well as factors in the WNT/β-catenin pathway, have been shown to be targets of miR-129 in NSCLC cell lines. Upregulation of miR-129, which was also found in BMs in the present study, could negatively affect EMT and the WNT/β-catenin pathway, which could negatively impact proliferation and especially metastatic potential of lung cancer cells [27]. miR-129 can also induce increased apoptosis and increased chemosensitivity by targeting SRY-box transcription factor 4 (*SOX4*) mRNA, as well as increased radiosensitivity through the silencing of *SOX4* and RUNX family transcription factor 1 (*RUNX1*) [28,29].

A target of miR-199, glucose regulated protein 78 (*GRP78*, also known as *HSPA5*), is the main regulator of the unfolded protein response (UPR) pathway and promotes proliferation, metastasis, and resistance to systemic therapy [30]. Downregulation of miR-199 could, therefore, increase tumor-promoting activities through the increased translation of *GRP78* [31]. miR-199a-5p has also been found to inhibit the signal transducer and activator of transcription 3 (STAT3) signaling pathway by targeting hypoxia inducible factor 1a (*HIF-1α*), an event which finally leads to decreased proliferation and migration, as well as increased apoptosis of NSCLC cells [32]. In the same study, HIF-1α/STAT3 axis suppressed the expression of miR-199a-5p, and a positive feedback loop which promotes the continuous progression of NSCLC was hypothesized [31]. *MAP3K11* was reported to be another target of miR-199a-5p in a study conducted in NSCLC patient tissues and mouse xenograft tumors. miR-199a-5p inhibited NSCLC cell proliferation by targeting *MAP3K11*, and as a result the MAPK pathway [33]. The observed downregulation of the miR-199 family in BM could, therefore, lead to increased proliferation and migration of the NSCLC cells. miR-150-5p has also been proposed as a tumor suppressor miRNA for squamous cell lung cancer, mostly by targeting genes related to the cell cycle [34]. Matrix metalloproteinase 14 (*MMP14*) was found to be another target of miR-150-5p in squamous cell lung cancer tissue biopsies. The upregulation of miR-150-5p can reduce cell migration and invasion by inhibiting *MMP14* expression [35]. The overexpression of several target genes and pathways, such as Liver Kinase B1 (*LKB1*), *HMGA2*, Wnt-β-catenin signaling pathway, and tensin 4 (*TNS4*) has been attributed to the downregulation of miR-150 and can lead to NSCLC tumor progression and metastasis [36,37,38]. However, there are some studies that have proposed miR-150 as a tumor-promoting miRNA (oncomiR), rendering its downregulation as an antitumoral cellular process. Proposed targets related to an oncogenic role of miR-150 are forkhead box O4 (*FOXO4*), BRI1-associated receptor kinase 1 (*BAK1*), Sirtuin 2/JmjC histone demethylase 2A (*SIRT2*/*JMJD2A*) signaling pathway, and v-src avian sarcoma viral oncogene homolog (SRC) kinase signaling inhibitor 1 (*SRCIN1*) [39,40,41,42]. miR-150 overexpression was also associated with higher risk for lymph node and distal metastasis, as well as shorter overall survival in a single institution Chinese study with 153 NSCLC patients [43]. miR-142-3p functions as a tumor suppressing miRNA by decreasing the expression of tumor-promoting genes such as high-mobility group box 1 (*HMGB1*), phosphatidylinositol-4,5-bisphosphate 3-kinase, catalytic subunit alpha (*PIK3CA*), Nuclear Receptor Subfamily 2 Group F Member 6 (*NR2F6*), and X-linked inhibitor of apoptosis protein gene (*XIAP*) [44,45,46,47]. In a study which correlated genome-wide miRNA expression profiles in a lung squamous cell carcinoma (LUSC) cohort (*n* = 57) with the Surveillance, Epidemiology, and End Results (SEER)-Medicare database, miR-142-3p overexpression was associated with good prognosis and chemosensitivity in NSCLC patients in all studied datasets [48].

The findings of the first part of our analyses regarding the upregulated miRNAs in the BM do not seem to be directly related to the aggressive nature of brain metastatic NSCLC, since almost all upregulated miRNAs are mainly reported to result in the suppression of tumor growth and invasiveness. On the other hand, the majority of published data related to the observed downregulated miRNAs in the BM in our study are consistent with the aggressiveness and dismal prognosis of brain metastatic NSCLC. However, the published data should be interpreted with caution. There are not enough clinical data that correlate the expression of miRNAs with the prognosis of NSCLC patients—the vast majority of these studies have been performed in vitro or in mouse models—and there are also contradictory results published for some miRNAs (e.g., miR-150).

In the second part of our analyses, we conducted miRNA target gene prediction by extracting all predicted target genes based on 3′UTR seed sequence complementarity, followed by a perturbation gene/pathway analysis model in order to further explore the molecular implications of the differential miRNA expression in the BM and primary tumor compartments. We used the impact analysis method that leverages the information about type, function, position, and interaction between genes in a given pathway. This novel type of analysis combines the evidence obtained from the classical enrichment analysis with a novel type of evidence, which measures the actual perturbation on a given pathway under a given condition [49].

When analyzing the cumulative effect of all differentially expressed miRNAs on potential target genes, *LCOR* emerged as the post-pronounced target gene. Interestingly, *LCOR* has been reported as a target of miR-199a in mammary stem cells and to influence initiation of ER-breast tumors [50]. However, it is difficult to assess the biological impact of a cumulative target unless all differentially expressed miRNAs display altered expression with identical trends. Therefore, we next separated our analysis into target genes of miRNAs upregulated in metastasis and target genes of miRNAs downregulated in metastasis. The impact analysis related to the miRNAs which were upregulated in brain metastasis revealed a core network of five target genes (*RANBP2*, *CLIP1*, *PPP2R5E*, *TAOK1*, and *DYNC1LI2*) predicted to be downregulated in brain metastasis. Three of these genes (*PPP2R5E*, *TAOK1*, and *DYNC1LI2*) were predicted to be upregulated in the analysis conducted for the downregulated miRNAs in the BM, and thus their role in the brain metastatic setting is difficult to interpret. The most regulatory connections, eight connections, were found for *CLIP1*. The fusion CLIP1-LTK, which affects around 0.4% of NSCLC patients, has recently been proposed as an oncogenic driver [51]. The predicted lower expression of *CLIP1* could, therefore, lead to decreased tumor activity, something which is in line with the predicted cellular effects of the upregulated miRNAs in the BM, as mentioned above.

Twelve target genes (*TP53*, *EP300*, *RAC1*, *XPO1*, *PPP2R5E*, *SKP1*, *TAOK1*, *DYNC1LI2*, *AGO1*, *MEF2C*, *FBXW11*, and *FBXO21*) were predicted to be upregulated in BM due to the downregulation of miRNAs. Some of these genes have been proposed as favorable and others as unfavorable prognostic biomarkers in different types of solid tumors [52,53]. The most regulatory connections, nine, were found for *TP53*, an important gene that suppresses carcinogenesis, which was found to be computationally predicted to be upregulated in BM [54]. The upregulation of *TP53* could lead to the suppression of tumor growth and invasiveness, albeit its role in the brain metastatic setting warrants more investigation [55,56]. It is difficult to interpret the potential impact of a transcriptional upregulation of *TP53* without knowing its mutational status on the DNA level since *TP53* mutants may promote metastasis, as previously described in breast cancer [56].

One of the limitations of our study is the absence of protein expression or mutation analysis, which could potentially strengthen the validity of our results. Another limitation is the absence of clinically relevant data regarding the biological effects of the miRNAs that were differentially expressed in the BM, which is something that renders the interpretation of our findings difficult. The interaction between the uncovered differentially expressed miRNAs has not been thoroughly investigated, which is imperative to explain how our miRNA signature could potentially play a role in the prognosis of NSCLC patients with BM. It should also be stressed that miRNAs are molecularly promiscuous players with each miRNA able to regulate hundreds of target genes. Given the molecular function of miRNAs, it is difficult to directly correlate our findings to previous reports investigating these miRNAs in distinct biological sources. In the second part of our study, we used an advanced perturbation pathway analysis, which takes into consideration the type, function, position, and interaction between genes in a given pathway. These results should also be interpreted with caution since they are not verified with a gene expression or mutation analysis.

In conclusion, we identified a unique miRNA signature in brain metastatic NSCLC samples compared with primary tumors, which could serve as biomarkers or potentially play a role in the dismal prognosis of NSCLC patients with BM.

## 4. Materials and Methods

### 4.1. Patient Cohort

The patient cohorts used for this study have been previously described [12]. The source population included 725 patients with surgically removed NSCLC. Of them, 280 patients received whole-brain radiotherapy during the course of their disease. Within this group, we identified patients with primary tumor and brain metastatic disease for systematic miRNA expression. The material was obtained from 25 patients with surgically removed BM, of which 14 underwent surgery of the primary tumor [12].

### 4.2. Sample Preparation

The sample preparation, including RNA extraction, was performed as previously described [12]. FFPE tissue sections of primary tumors, 4 × 4 μm, and matched brain metastases using RNeasy FFPE kit (Qiagen, Hilden, Germany) were utilized for the RNA isolation. RNA quantity and quality were assessed in all tissue samples using RNA Screen Tapes on a 2200 TapeStation system (Agilent, Santa Clara, CA, USA). Similar RNA integrity number curves were observed in all tissue samples.

### 4.3. miRNA Expression and Pathway Analysis

Systematic analysis of expression of 827 miRNAs was performed on the nCounter Human v3 miRNA Expression Assay (NanoString Technologies, Inc., Seattle, WA, USA) with a total of 100 ng of total RNA per sample as input. Scatter correlation plots and hierarchical clustering were visualized using normalized expression data in the nSolver analysis software (NanoString Technologies, Inc., Seattle, WA, USA) with raw counts normalized to five housekeeping genes. Euclidean distance with complete linkage was used for hierarchical clustering. Receiving Operating Characteristic (ROC) analysis was performed using GraphPad Prism software (GraphPad Software, San Diego, CA, USA).

In silico predicted target genes of upregulated miRNAs in BM (miR-129-2-3p, miR-124-3p, miR-219a-2-3p, miR-219a-5p, and miR-9-5p) and downregulated miRNAs in BM (miR-142-3p, miR-150-5p, miR-199b-5p, miR-199a-3p, miR-199b-5p, and miR-199a-5p) were extracted from TargetScanHuman 7 (Whitehead Institute for Biomedical Research, Cambridge, MA, USA) and ranked on their cumulative context scores. Context scores were transformed to RNA-sequencing expression values depending on the total number of miRNA to target gene interactions in each group multiplied with the individual context scores for each target gene. Target genes of upregulated miRNAs were considered as downregulated genes in BM. Target genes of downregulated miRNAs were considered as upregulated genes in BM.

Artificially transformed RNA-sequencing expression data (logFC values) were analyzed using Advaita Bioinformatics iPathwayGuide (iPG) (Advaita Corporation, Ann Arbor, MI, USA). iPG provides biological context and inferences from data generated by high-throughput sequencing and allows for the identification of miRNAs and putative mechanisms on the given differential expression signature. In this study, the miRNA activity was predicted through the enrichment of the differentially expressed target genes of the given miRNAs. The ratio between the absolute number of differentially downregulated gene targets of a given miRNA and all differentially expressed target genes was calculated via this software. Subsequently, the ratio of downregulated targets was compared with all targets for each miRNA. Finally, the probability of accidentally identifying an excess of differentially downregulated target genes was estimated. The final result was a list of target genes linked to their respective miRNAs ranked according to their *p*-value. Correction factors were adjusted for multiple comparisons. Pathway analysis as well as gene interaction hub network analysis was performed in iPG based on the final ranked miRNA target gene list. Hub network analysis was divided into target genes of miRNAs upregulated in BM and target genes of miRNAs downregulated in BM [49].

## Figures and Tables

**Table 1:** Baseline demographics and disease characteristics.

Variable	% (*n*)
Sex (male)	64 (16)
Age (mean ± SD)	62 ± 7.8
WHO Performance Status	
0–1	80 (20)
2	16 (4)
3	4 (1)
Histology (%)	
Adenocarcinoma	68 (17)
Squamous cell	16 (4)
SCLC	4 (1)
Large cell	12 (3)
CNS metastasis at diagnosis	24 (6)
Oncologic therapy	
Surgery primary tumor	52 (13)
Systemic therapy	80 (20)
CNS radiotherapy	92 (23)
GPA class group (%)	
1 (0–1)	28 (7)
2 (1.5–2.5)	56 (14)
3 (3–4)	16 (4)
RPA class (%)	
1	20 (5)
2	64 (16)
3	16 (4)
Number of CNS metastases (%)	
1	40 (10)
2–3	28 (7)
>3	32 (8)
Stage at diagnosis	
I	20 (5)
II	8 (2)
III	40 (10)
IV	32 (8)

RPA: Recursive partitioning analysis, GPA: graded prognostic assessment, CNS: central nervous system, SCLC: small-cell lung cancer, SD: standard deviation.

## Data Availability

Raw data from the miRNA expression profiling are available from the corresponding author upon request.

## Supplementary Materials

The following supporting information can be downloaded at: https://www.mdpi.com/article/10.3390/ijms24010193/s1.
