# Supplementary material for: Matched Analyses of Brain Metastases versus Primary Non-Small Cell Lung Cancer Reveal a Unique microRNA Signature
Source: Int J Mol Sci. 2022 Dec 22;24(1):193. doi: 10.3390/ijms24010193 (PMC9820685; doi:10.3390/ijms24010193)
Supplement: Supplementary file 1 [file ijms-24-00193-s001.zip › captions.pdf]

Figure S1: Differential miRNA expression in paired NSCLC tumors versus brain metastatic lesions;

Table S1: Differentially expressed miRNAs in primary NSCLC tumors versus brain metastatic lesions;

Table S2: Normalized counts and statistical analysis of differential miRNA expression in paired NSCLC tumors versus brain metastatic lesions;

Table S3: Target gene ranking of differentially expressed miRNAs ranked on their cumulative context score;

Table S4: Significantly altered pathways of target genes from all differentially expressed miRNAs;

Table S5: Interactions between differentially expressed miRNAs and core hub target genes in published literature.
