# Supplementary figures and images for: Matched Analyses of Brain Metastases versus Primary Non-Small Cell Lung Cancer Reveal a Unique microRNA Signature
Source: Int J Mol Sci. 2022 Dec 22;24(1):193. doi: 10.3390/ijms24010193 (PMC9820685; doi:10.3390/ijms24010193)

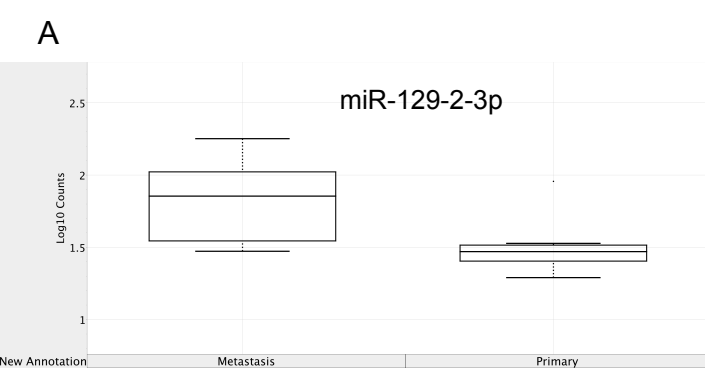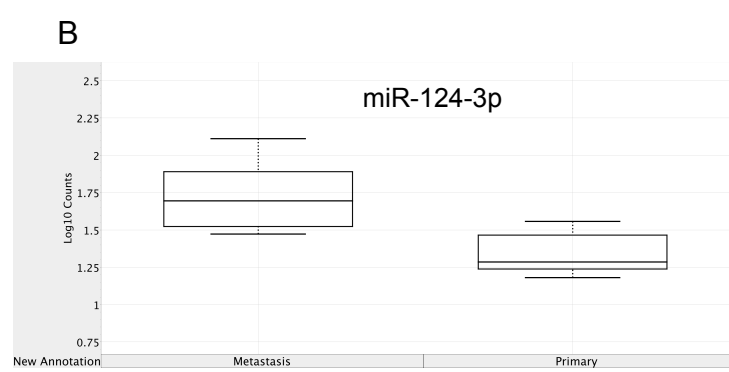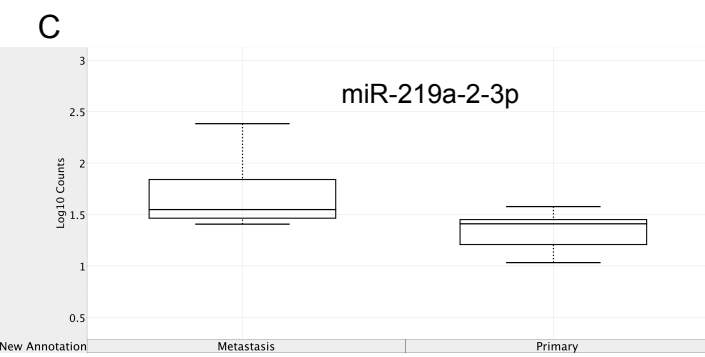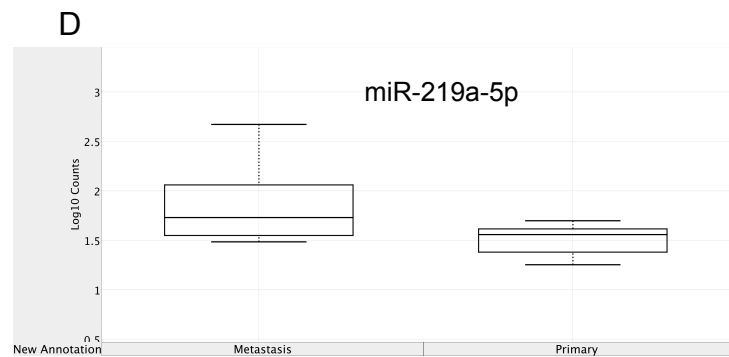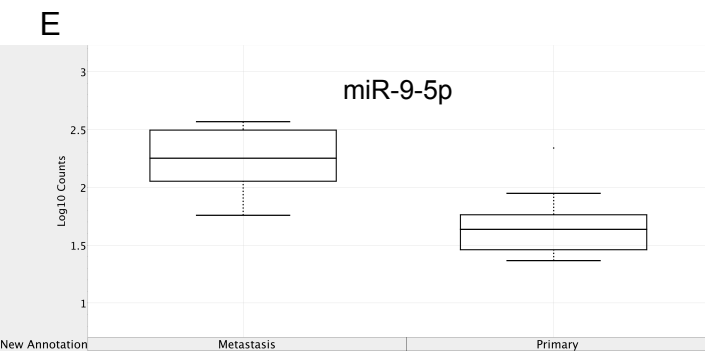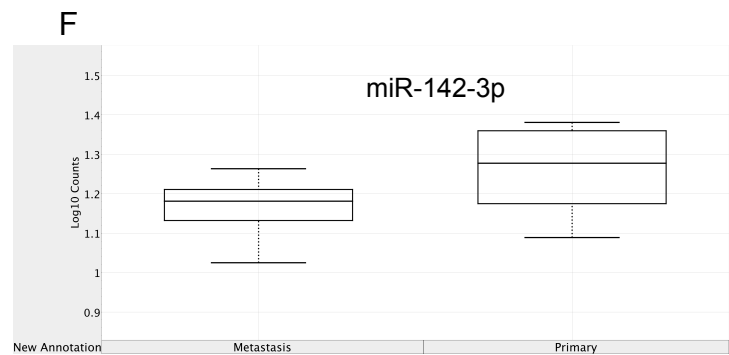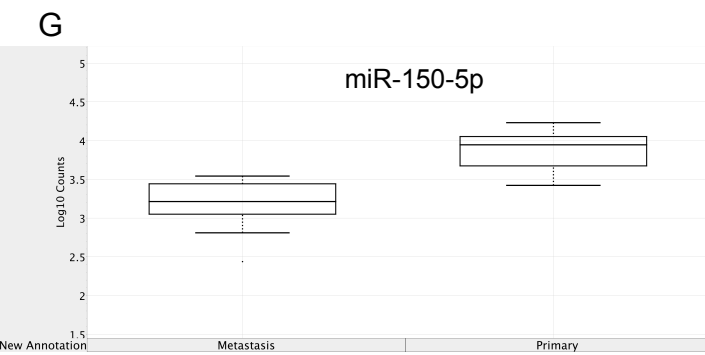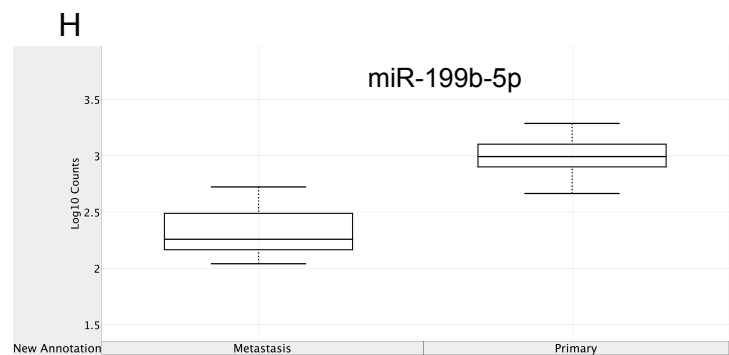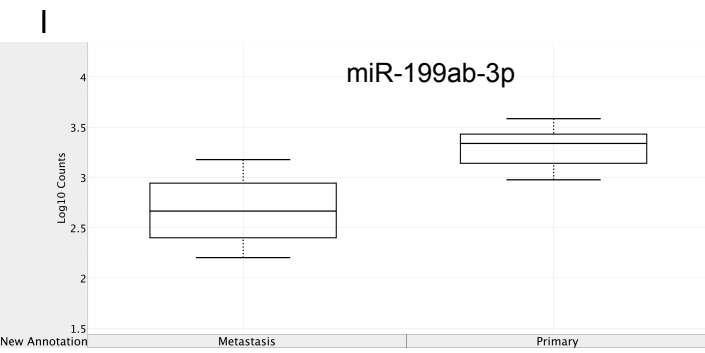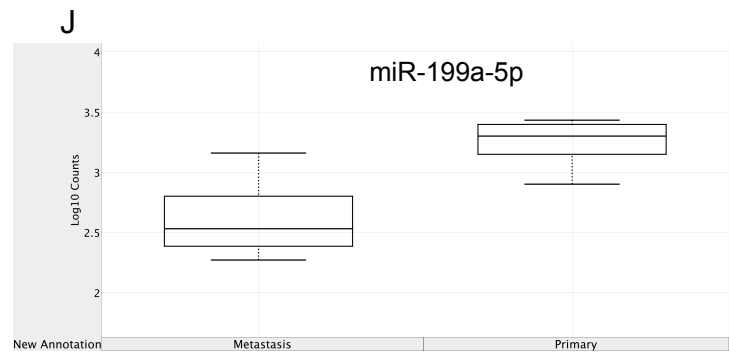

Supplement: Supplementary file 1 [file ijms-24-00193-s001.zip › Figure_S1.pdf]
